# Prognostic Value of Lactate Dehydrogenase in Second-Line Immunotherapy for Advanced Esophageal Squamous Cell Carcinoma

**DOI:** 10.3389/pore.2022.1610245

**Published:** 2022-06-03

**Authors:** Yan Li, Kunlun Wang, Erjiang Zhao, Bingxu Li, Shenglei Li, Xiaotao Dong, Ling Yuan, Hui Yang

**Affiliations:** ^1^ Department of Radiation Oncology, The Affiliated Cancer Hospital of Zhengzhou University & Henan Cancer Hospital, Zhengzhou, China; ^2^ Department of Biostatistics, The Affiliated Cancer Hospital of Zhengzhou University & Henan Cancer Hospital, Zhengzhou, China; ^3^ Department of Radiation Oncology, Anyang Tumour Hospital, Anyang, China

**Keywords:** immunotherapy, prognosis, esophageal squamous cell carcinoma, lactate dehydrogenase, programmed death -1

## Abstract

**Background:** Immunotherapy is recommended by the NCCN (National Comprehensive Cancer Network) guidelines as the standard second-line treatment for advanced esophageal squamous cell carcinoma (ESCC). Patients with advanced ESCC can benefit from immunotherapy, but the overall survival time (OS) is still not satisfactory. Therefore, it is of great importance to select effective prognostic indicators.

**Methods:** A retrospective follow-up study was conducted from January 2018 to January 2020 among 44 patients with advanced ESCC treated with second-line immune checkpoint inhibitors (programmed death -1 blocking agents) in our hospital. The cutoff values of baseline lactate dehydrogenase (LDH), LDH level at week 8, serum albumin, hemoglobin, neutrophils, monocytes, and platelets were obtained by receiver operating characteristic (ROC) curves. The Kaplan-Meier method was used to analyze the relationship between LDH at baseline, LDH level at week 8, and LDH changes during treatment with progression-free survival (PFS) and OS time. The Cox proportional hazards model was used for univariate and multivariate analyses to determine the predictors of OS.

**Results:** In univariate analysis, we found patients with lower baseline LDH levels (cutoff value: 200 U/L) had a better median PFS (8 months vs. 3 months; HR = 2.420, 95% CI: 1.178–4.971, *p* = 0.016) and OS (14 months vs. 6 months; HR = 3.637, 95% CI: 1.638–8.074, *p* = 0.004). The level of LDH at week 8 and the changes in LDH during treatment were not significantly associated with PFS or OS. The multivariate analyses showed that baseline LDH was an independent predictor of PFS (HR = 2.712, 95% CI: 1.147–6.409, *p* = 0.023) and OS (HR = 6.260, 95% CI: 2.320–16.888, *p* < 0.001), and the monocyte count (HR = 0.389, 95% CI: 0.162–0.934, *p* = 0.035) was significantly associated with OS.

**Conclusion:** Serum LDH is a powerful independent factor for PFS and OS in advanced ESCC patients treated with anti-PD-1 therapy.

## Background

Immunotherapy is the standard second-line treatment of advanced esophageal squamous cell carcinoma (ESCC) recommended by the National Comprehensive Cancer Network (NCCN) guidelines. Advanced ESCC patients can benefit from immunotherapy, but the overall survival (OS) time is still unsatisfactory. Therefore, it is important to select effective prognostic indicators to identify patient populations who are likely to benefit from immunotherapy. The elevation of lactic dehydrogenase (LDH) has been proved to predict the poor prognosis of various malignant tumors (1–3), such as pancreatic cancer, small-cell lung cancer, and melanoma. It plays an important role in glycolysis and inducing cell proliferation. Studies have confirmed that a high LDH level plays an important role in tumor metabolism, proliferation, invasion, and metastasis, and a high LDH level predicts a lower OS rate of cancer patients (4–7).

However, the prognostic role of LDH in the treatment of ESCC with immune checkpoint inhibitors (ICIs) has rarely been reported (8). We conducted a retrospective analysis of 43 ESCC patients and concluded that serum LDH is a potential marker of anti—programmed death -1 (PD-1) treatment and an independent factor affecting survival. The purpose of this study was to investigate the relationships among baseline LDH, week 8 LDH, and the changes in LDH during treatment and the prognosis of patients with advanced ESCC who received second-line anti-PD-1 immunotherapy, in order to identify new peripheral blood biomarkers.

## Data and Methods

### Patient Selection

This study retrospectively screened patients diagnosed in the Affiliated Cancer Hospital of Zhengzhou University from January 2018 to January 2020. The inclusion criteria were: advanced ESCC confirmed by pathology; clinical stage IV (according to tumor node metastasis [TNM] version 7); second-line treatment with ICIs (including camrelizumab, 25 patients; nivolumab, 1 patient; pembrolizumab, 6 patients; sintilimab, 2 patients); and Eastern Cooperative Oncology Group (ECOG) scores 0 or 1. Exclusion criteria of patients were as follows: concurrent or previous diagnosis of malignancy in other organs; autoimmune diseases; prior use of anti-PD-1, anti-PD-L1, or anti-PD-L2 drugs, or incomplete data.

### Clinical Data

We collected medical records from the hospital database, including patient age, gender, tumor location, previous smoking history, degree of differentiation, previous surgical treatment, previous radiotherapy, number of metastatic organs, week 8 LDH (U/L), and the changes in LDH during treatment. Pre-immunotherapy blood biomarkers included thyroid function, baseline LDH, hemoglobin, lymphocyte counts, mononuclear cell counts, platelet counts, serum albumin, and neutrophil counts.

### Treatment and Evaluation Criterion

PD-1 inhibitors are given intravenously as a single agent at an initial dose of 200 mg, repeated every 2–3 weeks, until disease progression, intolerable toxicity, or death.

Progressive disease (PD) refers to the increase in the sum of the two vertical diameters of the tumor by 25% above the lowest value or the emergence of a new tumor or other measurable diseases with significant progression.

Progression-free survival (PFS) is the period from the date of initial treatment with anti-PD-1 immunotherapy to the time of progression or death from any cause.

Overall survival (OS) is the time from the date of initial treatment with mAbs to death from any cause.

### Statistical Analysis

We performed blood tests on patients who were treated with immunotherapy. LDH levels were collected from all patients within 1 week before the first dose of PD-1 inhibitors and +3 days of subsequent doses. LDH levels were respectively divided into low vs. high levels at baseline and week 8 according to the cutoff of LDH at each stage. To differentiate patients with and without LDH change, and to indicate whether LDH levels decreased or increased between baseline and week 8, the population was divided into two categories: “decreased” (LDH difference was negative) and “increased” (LDH difference was zero or positive).

Receiver operating characteristic (ROC) curve analysis was used to determine the cutoff values.

A Kaplan–Meier survival curve was used to analyze the relationships of baseline LDH level, week 8 LDH level, and changes from LDH levels during treatment with PFS and OS. Univariate and multivariate analyses of predictive factors were performed by a Cox proportional hazards regression model. Proper factors with *p* < 0.1 in univariate analysis and other important factors were selected into multivariate analysis to validate independent prognostic factors. The results of prognostic factors were expressed as a hazard ratio (HR) with a 95% confidence interval (CI).

Throughout the analysis, *p* < 0.05 was considered statistically significant. SPSS Statistics software (version 25.0) was used for the analysis in the study.

## Results

Among the included 68 patients with advanced ESCC, some patients failed to complete immunotherapy due to intolerance; 3 patients were lost to follow-up, and 21 patients had no blood test results at the eighth week of treatment, so a total of 44 patients were ultimately included in the study. The end date of follow-up was August 2021, the median follow-up time was 13.80 months (range: 8.00 months–18.75 months), and 9 patients (20.50%) had survived. Median PFS was 6.00 months (95% CI: 5.40–9.78) and median OS was 11.00 months (95% CI: 10.45–15.67) in all patients. The characteristics of the study patients are shown in Table 1. A total of 77.30% of the study population were men; patients had a median age of 64.50 years (range: 57.00–69.75 years) at the time of diagnosis. The pathological differentiation degree of 68.20% of patients was good or moderate differentiation. A total of 65.90% of the patients had received previous radiation therapy, half of the patients had a history of smoking, and all the patients had metastasis to other organs. A total of 16 of the 44 patients (36.40%) had LDH levels greater than the cutoff at baseline, and 25 (56.82%) had increased LDH levels during treatment.

**TABLE 1:** Patient characteristics.

Characteristics	Total (*n* = 44)	Low LDH (*n* = 28)	High LDH (*n* = 16)
Age			
Median (range)	64.50 (57.00–69.75)	63 (55.25–68.75)	67 (57.00–72.00)
Gender			
Male	34 (77.30%)	22 (78.60%)	12 (75.00%)
Female	10 (22.70%)	6 (21.40%)	4 (25.00%)
Tumor location			
Cervical + upper	6 (13.60%)	4 (14.30%)	2 (12.50%)
Middle + lower	38 (86.40%)	24 (85.70%)	14 (87.50%)
Previous smoking history			
No	22 (50.00%)	13 (46.40%)	9 (56.25%)
Yes	22 (50.00%)	15 (53.60%)	7 (43.75%)
Degree of differentiation			
Poorly differentiated	14 (31.80%)	7 (25.00%)	7 (43.75%)
Well or moderately	30 (68.20%)	21 (75.00%)	9 (56.25%)
Previous surgical treatment			
No	34 (77.30%)	21 (75.00%)	13 (81.25%)
Yes	10 (22.70%)	7 (25.00%)	3 (18.75%)
Previous radiotherapy			
No	15 (34.10%)	10 (35.70%)	5 (31.25%)
Yes	29 (65.90%)	18 (64.30%)	11 (68.75%)
Number of metastatic organs			
≤2	36 (81.82%)	24 (85.71%)	12 (75.00%)
≥3	8 (18.18%)	4 (14.29%)	4 (25.00%)
Thyroid function			
Normal	16 (36.36%)	10 (35.71%)	6 (37.50%)
Abnormal	5 (11.36%)	1 (3.57%)	4 (25.00%)
Unknown	23 (52.28%)	17 (60.72%)	6 (37.50%)
PD-1 inhibitor			
Camrelizumab	25 (56.82%)	17 (60.72%)	8 (50.00%)
Nivolumab	1 (2.27%)	1 (3.57%)	0 (0.00%)
Pembrolizumab	6 (13.64%)	3 (10.71%)	3 (18.75%)
Sintilimab	12 (27.27%)	7 (25.00%)	5 (31.25%)
Week 8 LDH(U/L)			
<351	36 (81.82%)	27 (96.43%)	9 (56.25%)
≥351	8 (18.18%)	1 (3.57%)	7 (43.75%)
LDH change			
Decreased	19 (43.18%)	11 (39.29%)	8 (50.00%)
Increased	25 (56.82%)	17 (60.71%)	8 (50.00%)
HB(g/L)			
<130	30 (68.18%)	20 (71.43%)	10 (62.50%)
≥130	13 (29.55%)	8 (28.57%)	5 (31.25%)
Unknown	1 (2.27%)	0 (0.00%)	1 (6.25%)
Lym count (10^9^/L)			
<1.35	15 (34.09%)	10 (35.71%)	5 (31.25%)
≥1.35	28 (63.64%)	18 (64.29%)	10 (62.50%)
Unknown	1 (2.27%)	0 (0.00%)	1 (6.25%)
MONO count (10^9^/L)			
<0.315	25 (56.82%)	17 (60.71%)	8 (50.00%)
≥0.315	18 (40.91%)	11 (39.29%)	7 (43.75%)
Unknown	1 (2.27%)	0 (0.00%)	1 (6.25%)
PLT count (10^9^/L)			
<258.5	35 (79.55%)	23 (82.14%)	12 (75.00%)
≥258.5	9 (20.45%)	5 (17.86%)	4 (25.00%)
ALB count(g/L)			
<40	7 (15.91%)	4 (14.29%)	3 (18.75%)
≥40	36 (81.82%)	23 (82.14%)	13 (81.25%)
Unknown	1 (2.27%)	1 (3.57%)	0 (0.00%)
NE coun t (10^9^/L)			
<2.305	15 (34.09%)	10 (35.71%)	5 (31.25%)
≥2.305	28 (63.64%)	18 (64.29%)	10 (62.50%)
Unknown	1 (2.27%)	0 (0.00%)	1 (6.25%)

ALB, Serum albumin; HB, hemoglobin; NE, neutrophils; Lym, Lymphocytes; MONO, monocytes; PLT, platelets.

### Cutoff Values of Blood Biomarkers

ROC curves were generated to determine the cutoff values of blood biomarkers. The cutoff values for baseline LDH, the eighth week of LDH, serum albumin, hemoglobin, neutrophil counts, lymphocyte counts, monocyte counts, and platelet counts were 200.000, 351.000, 40.000, 130.000, 2.305, 1.350, 0.315, and 258.500, respectively (Table 2). Patients were divided into two groups based on the corresponding cutoff values.

**TABLE 2:** The cutoff values in ROC curve analysis.

Variables	Baseline LDH	The eighth week of LDH	ALB	HB	NE	Lym	MONO	PLT
Cutoff values	200.000(U/L)	351.000(U/L)	40.000(g/L)	130.000(g/L)	2.305 (10^9^/L)	1.350 (10^9^/L)	0.315 (10^9^/L)	258.500 (10^9^/L)

ALB, Serum albumin; HB, hemoglobin; NE, neutrophils; Lym, Lymphocytes; MONO, monocytes; PLT, platelets.

### Univariate Analysis of PFS and OS

**FIGURE 1:**
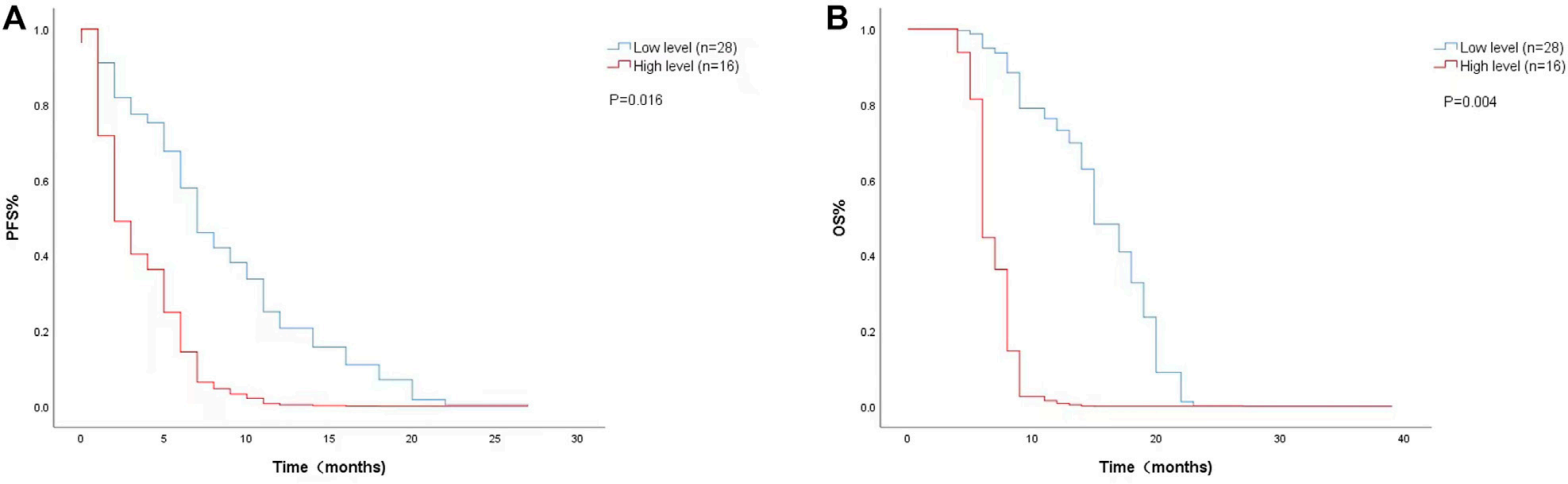
Kaplan-Meier curves of **(A)** PFS and **(B)** OS according to baseline LDH levels. (Low level <200 U/L (*n* = 28), high level ≥200 U/L (*n* = 16).

**FIGURE 2:**
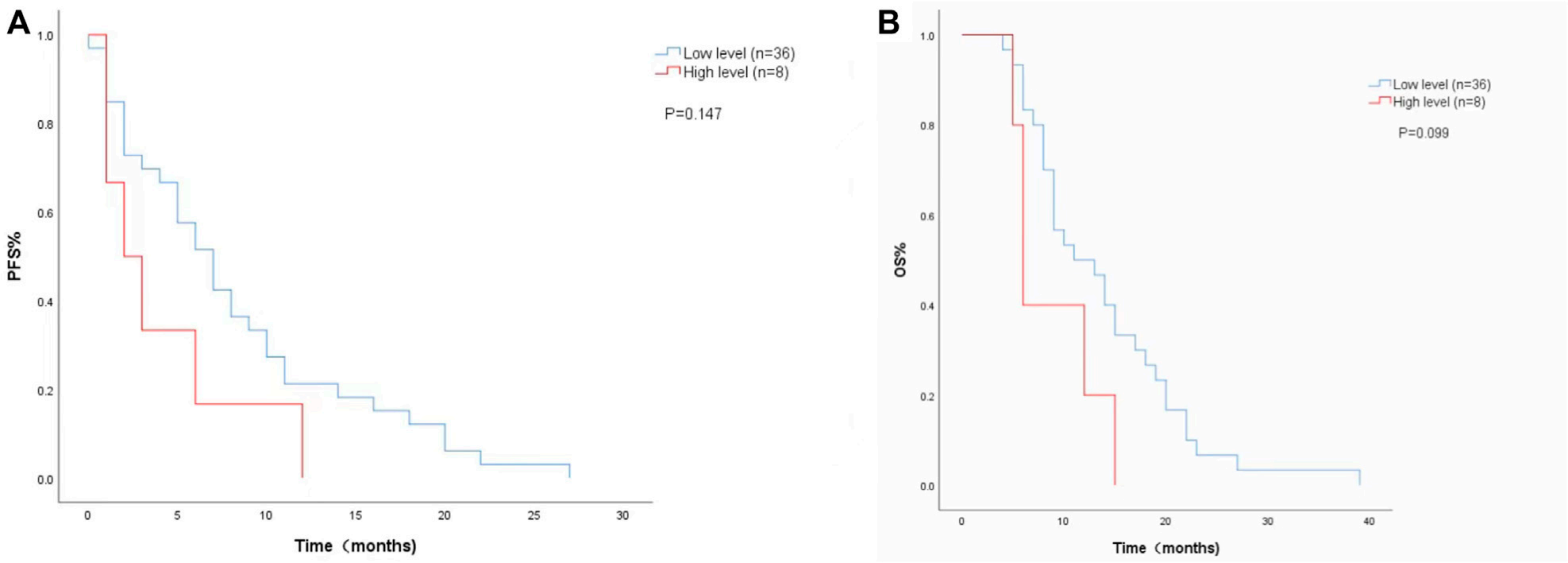
Kaplan-Meier curves of **(A)** PFS and **(B)** and OS according to LDH levels at week 8. (Low level <351 U/L (*n* = 36), high level ≥351 U/L (*n* = 8).

The Cox proportional risk model was used for univariate analysis, as shown in Table 3. In univariate analysis, we found patients with lower baseline LDH levels (cutoff value: 200 U/L) had a better median PFS (8 months vs. 3 months; HR = 2.420, 95% CI: 1.178–4.971, *p* = 0.016) (Figure 1A) and OS (14 months vs. 6 months; HR = 3.637, 95% CI: 1.638–8.074, *p* = 0.004) (Figure 1B). PFS and OS were assessed based on the week 8 LDH levels (cutoff value: 351 U/L), we found patients with lower LDH levels showed a median PFS (7 months vs. 2 months; HR = 1.940, 95% CI: 0.792–4.752, *p* = 0.147) (Figure 2A) and OS (11 months vs. 6 months; HR = 2.296, 95% CI: 0.856–6.161, *p* = 0.099) according to univariate analysis (Figure 2B).

**TABLE 3:** Prognostic factors of OS and PFS by Cox survival analyses.

		OS		PFS	
	N	HR (95%CI) (±95% CI)	*p* value	HR (95%CI)	*p* value
Age (year)					
<60	18				
≥60	26	1.433 (0.679–2.947)	0.326	1.363 (0.691–2.686)	0.371
Gender					
Male	34				
Female	10	0.656 (0.293–1.470)	0.175	1.254 (0.574–2.738)	0.570
Tumor location					
Cervical + upper	6				
Middle + lower	38	0.360 (0.118–1.094)	0.072	0.503 (0.148–1.711)	0.271
Previous smoking history					
No	22				
Yes	22	1.272 (0.629–2.575)	0.568	1.339 (0.704–2.545)	0.373
Degree of differentiation					
Poorly differentiated	14				
Well or moderately differentiated	30	0.571 (0.268–1.215)	0.142	0.711 (0.357–1.419)	0.334
Previous surgical treatment					
No	34				
Yes	10	1.637 (0.759–3.531)	0.205	1.164 (0.583–2.321)	0.667
Previous radiotherapy					
No	15				
Yes	29	1.669 (0.766–3.683)	0.193	1.603 (0.802–3.205)	0.182
Number of metastatic organs					
≤2	36				
≥3	8	1.519 (0.524–4.408)	0.438	1.651 (0.628–4.341)	0.309
thyroid function					
Normal	16				
Abnormal	5	1.916 (0.612–6.005)	0.264	1.458 (0.518–4.110)	0.475
Unknown	23	1.012 (0.491–2.088)	0.974	0.932 (0.462–1.880)	0.844
PD-1 inhibitor					
Camrelizumab	25				
Nivolumab	1	0.465 (0.060–3.626)	0.465	3.909 (0.486–31.444)	0.200
Pembrolizumab	6	2.022 (0.772–5.298)	0.152	1.997 (0.771–5.172)	0.154
Sintilimab	12	2.094 (0.934–4.692)	0.108	1.297 (0.614–2.742)	0.496
Baseline LDH (U/L)					
<200	28				
≥200	16	3.637 (1.638–8.074)	**0.004**	2.420 (1.178–4.971)	**0.016**
Week 8 LDH (U/L)					
<351	36				
≥351	8	2.296 (0.856–6.161)	0.099	1.940 (0.792–4.752)	0.147
LDH change					
Decreased	19				
Increased	25	0.789 (0.399–1.558)	0.495	1.151 (0.585–2.266)	0.684
HB(g/L)					
<130	30				
≥130	13	0.633 (0.284–1.410)	0.263	0.783 (0.384–1.599)	0.503
Unknown	1	1.461 (0.192–11.124)	0.714	0.724 (0.097–5.410)	0.753
Lym count (10^9^/L)					
<1.35	15				
≥1.35 9/L	28	0.606 (0.258–1.425)	0.251	0.975 (0.468–2.032)	0.947
Unknown	1	1.499 (0.198–11.373)	0.695	0.776 (0.104–5.798)	0.805
MONO count (10^9^/L)					
<0.315	25				
≥0.315	18	0.585 (0.290–1.199)	0.144	0.989 (0.511–1.915)	0.974
Unknown	1	1.339 (0.175–10.260)	0.778	0.778 (0.103–5.881)	0.808
PLT count (10^9^/L)					
<258.5	35				
≥258.5	9	0.965 (0.392–2.375)	0.939	1.024 (0.465–2.251)	0.953
ALB count (g/L)					
<40	7				
≥40	36	1.475 (0.562–3.868)	0.430	2.055 (0.845–4.994)	0.112
Unknown	1	2.297 (0.256–20.605)	0.457	1.764 (0.206–15.090)	0.604
NE count (10^9^/L)					
<2.305	15				
≥2.305	28	0.764 (0.361–1.618)	0.482	1.311 (0.673–2.554)	0.427
Unknown	1	1.355 (0.168–10.941)	0.775	0.916 (0.119–7.068)	0.933

Bold values mean *p* values <0.05. In all analyses, the first group was the reference group.

ALB, Serum albumin; HB, hemoglobin; NE, neutrophils; Lym, Lymphocytes; MONO, monocytes; PLT, platelets.

**FIGURE 3:**
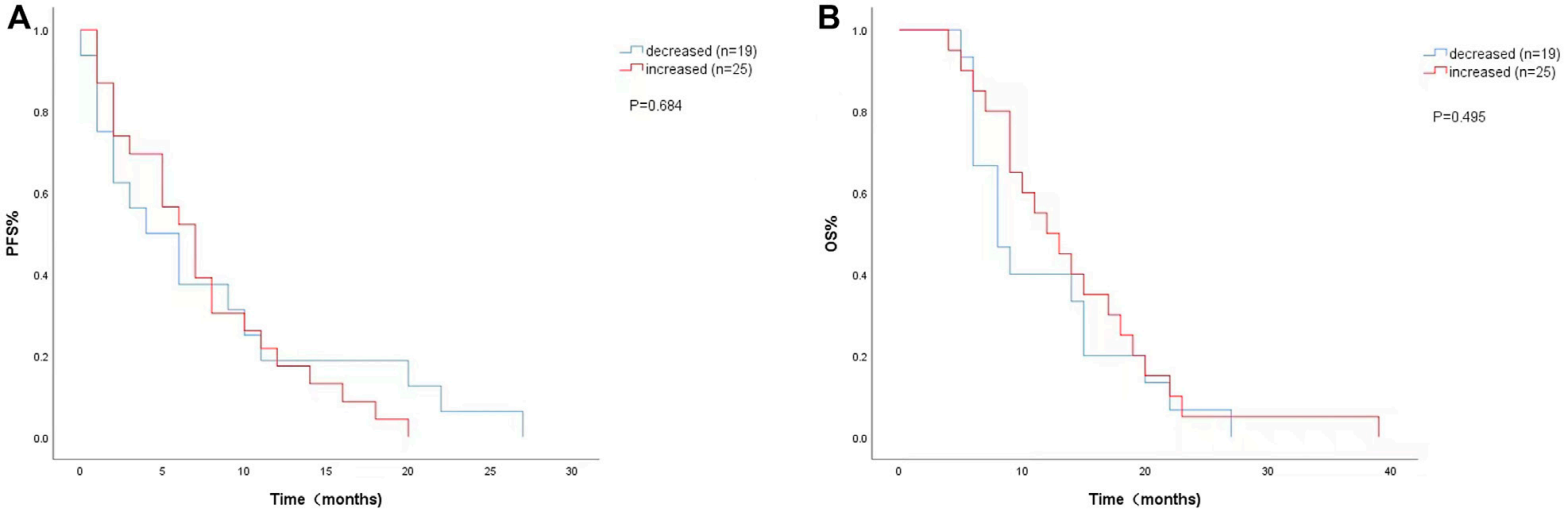
Kaplan-Meier curves of **(A)** PFS and **(B)** OS according to LDH changes during. treatment. (Decreased (*n* = 19): LDH difference was negative. Increased (*n* = 25): LDH difference was zero or positive).

The univariate analysis and Kaplan-Meier survival curve showed a median PFS (4 months vs. 7 months; HR = 1.151, 95% CI: 0.585–2.266, *p* = 0.684) (Figure 3A) and OS (8 months vs. 12 months; HR = 0.789, 95% CI:0.399–1.558, *p* = 0.495) (Figure 3B) with decreased LDH levels during treatment. The level of LDH at week 8 and the changes in LDH during treatment were not significantly associated with PFS or OS.

In addition, we considered that age, tumor location, monocyte count, hemoglobin, and the number of metastatic organs were also correlative to PFS and OS, so they were selected into multifactor analysis.

### Multivariate Analysis of PFS and OS

The multivariate analyses showed that baseline LDH was an independent predictor of PFS (HR = 2.712, 95% CI: 1.147–6.409, *p* = 0.023) and OS (HR = 6.260, 95% CI: 2.320–16.888, *p* < 0.001), and the monocyte count (HR = 0.389, 95% CI: 0.162–0.934, *p* = 0.035) was significantly associated with OS (Table 4).

**TABLE 4:** Multivariate analysis of the correlation between baseline patient characteristics and overall cohort patient survival (*n* = 44).

	N	OS		PFS	
		HR (95% CI) (±95% CI)	*p* value	HR (95% CI)	*p* value
Age (year)					
<60	18				
≥60	26	1.332 (0.591–3.000)	0.489	1.064 (0.498–2.272)	0.872
Tumor location					
Cervical + upper	6				
Middle + lower	38	0.319 (0.091–1.123)	0.075	0.590 (0.160–2.178)	0.428
Number of metastatic organs					
≤2	36				
≥3	8	1.976 (0.578–6.761)	0.278	1.533 (0.511–4.592)	0.446
Baseline LDH(U/L)					
<200	28				
≥200	16	6.260 (2.320–16.888)	**0.000**	2.712 (1.147–6.409)	**0.023**
Week 8 LDH(U/L)					
<351	36				
≥351	8	1.933 (0.472–7.913)	0.359	0.813 (0.233–2.842)	0.746
HB(g/L)					
<130	30				
≥130	13	0.603 (0.227–1.602)	0.310	0.804 (0.336–1.926)	0.624
Unknown	1	0.341 (0.038–3.059)	0.337	0.334 (0.036–3.097)	0.335
Lym count (10^9^/L)					
<1.35	15				
≥1.35 9/L	28	1.317 (0.470–3.689)	0.600	1.022 (0.436–2.396)	0.960
Unknown	1	0.341 (0.038–3.059)	0.337	0.334 (0.036–3.097)	0.335
MONO count (10^9^/L)					
<0.315	25				
≥0.315	18	0.389 (0.162–0.934)	**0.035**	0.758 (0.314–1.830)	0.538
Unknown	1	0.341 (0.038–3.059)	0.337	0.334 (0.036–3.097)	0.335

Bold values mean *p* values <0.05. In all analyses, the first group was the reference group.

ALB, Serum albumin; HB, hemoglobin; NE, neutrophils; Lym, Lymphocytes; MONO, monocytes; PLT, platelets.

## Discussion

The results showed that a low baseline LDH level brings on better PFS and OS than a high baseline LDH level during second-line immunotherapy for advanced ESCC. The changes from LDH levels during treatment and week 8 LDH levels did not show a significant association with PFS or OS. Baseline LDH has an independent predictive value for the outcome of immunotherapy for advanced ESCC. The reasons why serum LDH levels can be regarded as a predictor of tumors can be concluded as follows. From a metabolic perspective, regardless of a normoxic or hypoxic environment, malignant tumor cells are in an active state of glycolysis, and the production of lactic acid is enhanced because of the so-called Warburg effect (9). LDH is the catalyst for the conversion of pyruvate into lactic acid during glycolysis, so the level of LDH will increase with the enhancement of glycolysis in tumor cells. In addition, LDH is thought to be an indicator of tissue breakdown, in cancer patients, the cancer cell cycle is shortened due to the strong ability of the cells to proliferate, leading to an increased risk of necrosis. Moreover, adjacent normal tissues such as the lung, liver, and bone may be invaded by cancer cells (1,10), and the damage to these organs will also cause the LDH level to rise. There are also studies that show that high LDH levels may lead to lactic acid production and acidification of the extracellular water space, which contributes to increased invasion of cancer cells (11). In these senses, LDH can be considered a housekeeping enzyme released by rapidly growing tumors, and it is also closely related to tumor invasion and metastasis. All of these mechanisms may jointly promote the elevation of serum LDH levels in cancer patients, making it a possible predictor of tumor prognosis.

Studies on whether LDH can predict survival have also been reported for other cancers. Corine DeJong et al.(12) retrospectively analyzed 593 patients with advanced non-small-cell lung cancer (NSCLC) who received first-line platinum-based chemotherapy. They found that a reduction in LDH, especially early in treatment, was significantly associated with a better radiation response; a higher LDH level at baseline was significantly associated with lower OS. A meta-analysis of the predictive role of LDH in ICI-treated NSCLC patients showed that a high pretreatment LDH level was significantly associated with poor prognosis in ICI-treated NSCLC patients (2). Our findings are in line with these published findings. There are few studies on the prognostic value of LDH in ESCC, the largest study to examine the prognostic value of LDH in ESCC was a retrospective study on the OS of 906 patients with ESCC, the results showed that a high level of LDH was associated with TNM stage and distant metastasis, and the survival time of patients with a high level of LDH was shorter (13). Similarly, our results in advanced ESCC immunotherapy patients showed that patients with high LDH at baseline had shorter PFS and OS than patients with low LDH at baseline. Most previous studies have focused only on baseline values, with little attention being paid to whether dynamic changes in LDH during treatment are associated with patient outcomes (1,2). Our results not only showed that lower LDH levels at baseline were associated with better PFS and OS but also found that changes in LDH during treatment had no effect on patient outcomes. Studies have shown that neutrophils, lymphocytes, and hemoglobin are correlated with the survival rate of patients and can be used as prognostic markers of treatment (14–16). However, they did not show a significant association with PFS or OS in our study, which may have been caused by the small sample size. Monocyte count can be used as a predictor, possibly because monocytes may have protumor effects by recruiting neutrophils to the tumor microenvironment by secreting interleukin-10 (IL-10) to achieve immunosuppressive effects (17). Monocytes can also promote angiogenesis, leading to the rapid progression of cancer (18), and their role in tumor prediction has been confirmed (19).

Our study has several limitations. First, it was a single-center retrospective study. Second, the number of patients included was relatively small, and the follow-up time was short. Future studies with a larger sample size are needed.

A study has shown that for patients who are designated for chemotherapy, tackling elevated LDH levels before treatment may alleviate tumor stress and improve the efficacy of chemotherapeutic agents, thus gaining a survival benefit in the end (1). This may be related to the fact that the inhibition of LDH expression can reduce the invasion and metastatic potential of cancer cells by reducing their proliferation ability and reversing their resistance to chemotherapy (20). Han Xie et al.(21) used a newly developed inducible mouse model to inactivate LDH-A, which was shown to lead to reduced tumor occurrence and regression, further indicating that suppressing LDH can benefit cancer patients. LDH measurement plays a key role in monitoring the effect of immunotherapy on patients with advanced ESCC, and it can be obtained through a simple blood test. So it has the advantages of being rapid, inexpensive, and convenient for clinical application. Therefore, its prognostic value deserves further study and wider application.

## Data Availability

The original contributions presented in the study are included in the article/supplementary material, further inquiries can be directed to the corresponding authors.
